# Connecting Diverse Knowledge Systems for Enhanced Ecosystem Governance: The Multiple Evidence Base Approach

**DOI:** 10.1007/s13280-014-0501-3

**Published:** 2014-03-22

**Authors:** Maria Tengö, Eduardo S. Brondizio, Thomas Elmqvist, Pernilla Malmer, Marja Spierenburg

**Affiliations:** 1 Stockholm Resilience Centre, Stockholm University, Kräftriket 2A, 106 91 Stockholm, Sweden; 2 Department of Anthropology, Indiana University, Student Building 130, Bloomington, IN 47405 USA; 3 The Resilience and Development Programme – SwedBio, Stockholm Resilience Centre, Stockholm University, Kräftriket 2A, 106 91 Stockholm, Sweden; 4 Department of Organization Sciences, VU University Amsterdam, De Boelelaan 1081, 1081 HV Amsterdam, The Netherlands

**Keywords:** Local knowledge, Indigenous knowledge, Complementarity, Validation, Ecosystem assessments, Co-production of knowledge

## Abstract

Indigenous and local knowledge systems as well as practitioners’ knowledge can provide valid and useful knowledge to enhance our understanding of governance of biodiversity and ecosystems for human well-being. There is, therefore, a great need within emerging global assessment programs, such as the IPBES and other international efforts, to develop functioning mechanisms for legitimate, transparent, and constructive ways of creating synergies across knowledge systems. We present the multiple evidence base (MEB) as an approach that proposes parallels whereby indigenous, local and scientific knowledge systems are viewed to generate different manifestations of knowledge, which can generate new insights and innovations through complementarities. MEB emphasizes that evaluation of knowledge occurs primarily within rather than across knowledge systems. MEB on a particular issue creates an enriched picture of understanding, for triangulation and joint assessment of knowledge, and a starting point for further knowledge generation.

## Introduction

Ecosystem processes interact with and enrich human lives, contributing to economies and human well-being in a wide range of ways. How we can sustainably manage and govern the ecosystems on which we depend is a tremendously complex challenge. To succeed, we cannot afford to lose insights and information originating from multiple knowledge systems.^1^ The rapid acceleration and intensity of global environmental change places great demands on humanity to develop innovative ways and processes for connecting knowledge systems that are conducive to sustainability learning and recognize the complexities of social–ecological systems and the challenges of the Anthropocene (Berkes et al. 2003; Folke et al. 2011; Tàbara and Chabay 2012). Indigenous and local knowledge systems, developed through experimentation, adaptation, and co-evolution over long periods of time can provide valid and useful knowledge, as well as methods, theory and practices for sustainable ecosystem management.^2^ Together with the natural and social sciences they can enhance our understanding of how to care for and strengthen the role of biodiversity and ecosystems for human well-being (Reid et al. 2006; Turnhout et al. 2012; Thaman et al. 2013).

In many regions, and for many aspects of governance in social–ecological systems, our sole source of knowledge may reside among local users and managers. One example is climate change in the tropics and the role of agrobiodiversity for adapting to variability and sustaining local livelihoods (Mijatović et al. 2013). Local people across the globe are continuously faced with the challenges of adapting and developing their knowledge to cope with local manifestations of regional and global environmental change. Part of this knowledge is locally or regionally maintained, adapted, and transmitted both orally and in practice, but is also in constant interaction with other forms of knowledge (Berkes 2008; Nakashima et al. 2012). Recognizing and strengthening existing systems for learning and for responding with experience to change and novel conditions is essential for building resilience (Berkes and Folke 2002). Furthermore, cross-fertilization among a diversity of knowledge systems can contribute new evidence and also improve the capacity to interpret conditions, change, responses, and in some cases causal relationships in the dynamics of social–ecological systems. Further, it may also lead to innovation and the identification of desirable trajectories or pathways into the future.

The academic literature provides examples from across the globe where the recognition of complementarities across knowledge systems have advanced the understanding, and in many cases improved management, of ecosystems, critical natural resources, and biodiversity. They include for instance the understanding of sea ice dynamic and climate change (Laidler 2006), population dynamics of fish and other wildlife (Mackinson 2001; Moller et al. 2004; Gagnon and Berteaux 2009; Prado et al. 2013), as well as land use change and farming practices (Brookfield et al. 2003; Chalmers and Fabricius 2007; Brondizio 2008) (see more details in Table 1). While this potential is increasingly acknowledged in science and policy spheres, to date there has been limited success in bringing knowledge systems together in assessments and international science-policy processes (but see Danielsen et al. (2014) for an assessment of potential, and Weismann and Hurni (2011) for experiences from sustainability science).

**Table 1:** Examples of case studies using a parallel approach to connecting knowledge systems

Issue investigated	Multiple evidence base	Reflections on scale and complementarity
Relationship between Arctic sea ice and climate change (Laidler 2006)	Literature review assessing current research presenting Inuit knowledge or observations of sea ice, along with scientific knowledge or observations of sea ice	Inuit knowledge at local (*mainly at fine scales*) and regional, spanning living memory to the past, through historical recall. Scientific knowledge at local, regional, and global (*mainly at coarse scales*), and short time depth
Monitoring for sustainable customary wildlife harvests in Canada and New Zealand (Moller et al. 2004)	Data sharing and calibrating traditional monitoring methods against scientific abundance measures. Interviews and collaborations with hunters	Local knowledge: add long time periods, larger samples, extreme events and adaptive strategies, and sometimes multivariate cross-checks for environmental change Scientific knowledge: better tests of potential causes of change on larger spatial change, precise quantification, and evaluation without harvesting
Land use and land cover change and underlying drivers, Wild Coast, Eastern Cape, South Africa (Chalmers and Fabricius 2007)	Comparing local and scientific understanding based on interviews with local experts and other local representatives, and reviewing scientific literature on forest-savannah dynamics	Local experts added detailed understanding of ultimate causes of change, how drivers interact, and adding historical perspectives interacting at multiple temporal and spatial scales Scientific knowledge was more coarse grained and added perspectives of causal mechanisms and an ability to study and predict obscure processes such as the impact of atmospheric change on vegetation
Fish population spatial dynamics, British Columbia, Canada (Mackinson 2001)	Combining knowledge of fish behavior and distribution. Interviews with fishery scientists, fishery managers, and local fishers	Local fishers provided in-depth and detailed information from observation, but were generally reluctant to interpret or rank the data. In combining the three sources, there were no instances in which knowledge opposed another or diverged from that found in scientific literature
Ecology of Arctic Fox and Snow Goose in Nunavut, Canada (Gagnon and Berteaux 2009)	Investigating the complementarity of Inuit TEK and scientific knowledge across spatial and temporal scales. Workshops, interviews, mapping for collecting TEK, review of scientific information	Complementarity in temporal (e.g., winter feeding ecology) and spatial (e.g., feeding ranges) scales in understanding across traditional ecological knowledge and scientific knowledge, more expressed for Arctic fox than Snow goose
Agroforestry intensification in the Amazon estuary (Brondizio 2008)	Investigation involved learning from and doing experiments with estuarine small farmers on the management techniques used to intensify food production (acai palm fruit) without deforestation. Historical remote sensing and quantitative data complements ethnography and participant observation, ethnobotany and household surveys	Local farmers demonstrated techniques of forest management and agroforestry intensification in different parts of the landscape. Historically considered as passive extractivists of forests, collaboration has allowed to demonstrate the sophistication local food production systems in forest areas, to question established misconceptions of native farmers as backward and irrelevant to the regional economy, and to show how local knowledge has allowed the acai palm fruit to become a global product without causing local deforestation
The effect of free-ranging domestic reindeer grazing on biodiversity and vice versa in Northern Sweden (Tunón and Sjaggo 2012)	Combining scientific knowledge of the impact on reindeer herding on biodiversity with reindeer herder’s perspectives on the role of biodiversity for the reindeer management and landscape change	Herder’s knowledge adding landscape-level insights time depth, the role of additional biotopes for herding, and the management perspective connecting different biotopes in time and space. Scientific knowledge focus on high-resolution, small scale studies with a short time depth

There is a great need to develop mechanisms to engage in legitimate, transparent, and constructive ways of creating synergies across knowledge systems (Reid et al. 2006; Turnhout et al. 2012). This is visible and embedded in the goals of many international efforts, from the on-going proposals for the new sustainable development goals (SDG; e.g., to improve food security and decrease local vulnerability to environmental change) to the Aichi targets of the Convention on Biological Diversity (CBD 2013a). In fact, the recently established Intergovernmental Science-Policy Platform on Biodiversity and Ecosystem Services (IPBES) prominently features the recognition and respect for indigenous and local knowledge and its contribution to the conservation and sustainable use of biodiversity and ecosystems as part of its operational principles (IPBES 2012), as well as in the assessment agenda and work program (IPBES 2013a; Thaman et al. 2013). There is a great opportunity for contributing to the efforts of the IPBES to develop frameworks that can enable synergies between knowledge systems in its work (Turnhout et al. 2012; Sutherland et al. 2013), which would also benefit other initiative such as the SDGs and the goals of the CBD (CBD 2013a). In this paper, we present a first step to outlining such an approach, the *multiple evidence base (MEB)*, with the aim of stimulating discussion among all actors involved and contributing to a useful pathway ahead.

**Fig. 1:**
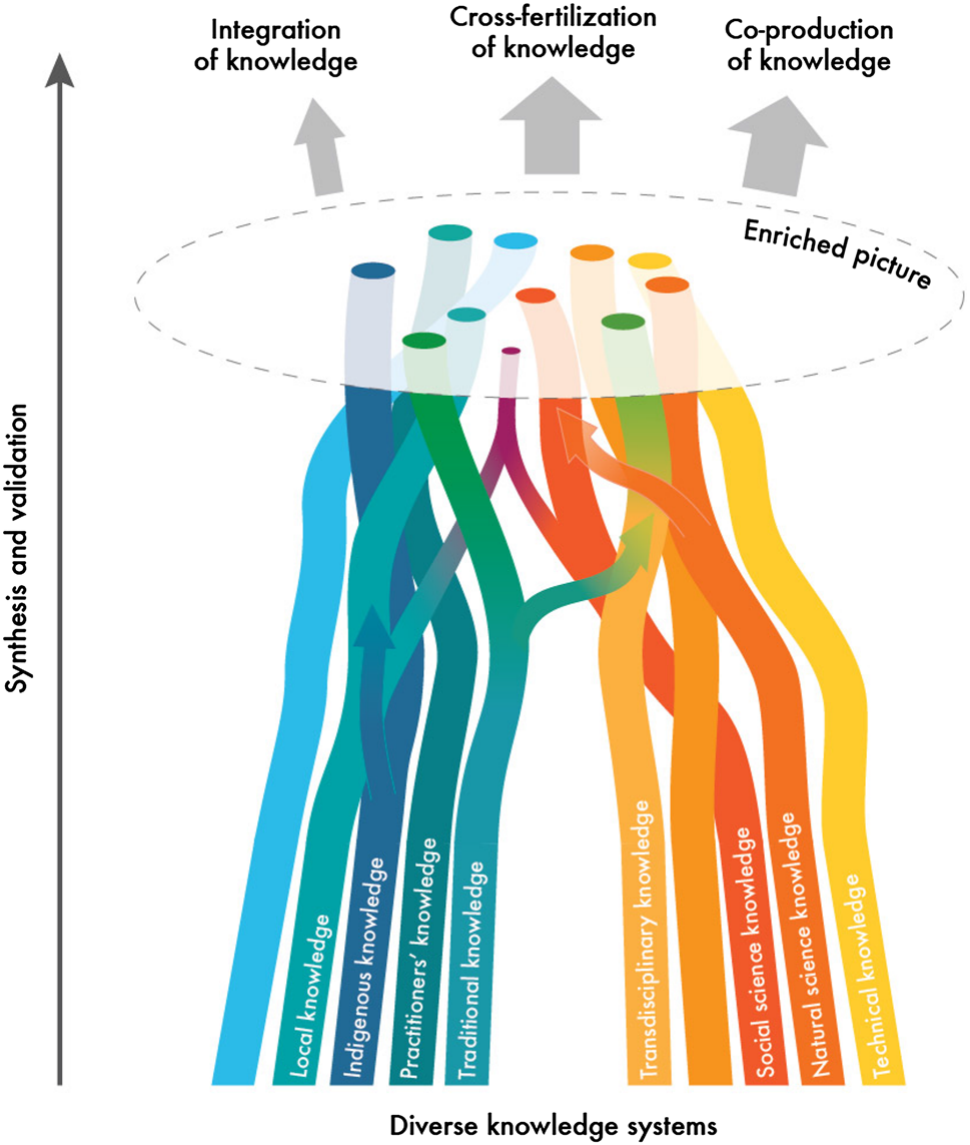
An illustration of a multiple evidence base approach, where diverse knowledge systems contribute to generate an enriched picture of a selected problem or issue of concern. The enriched picture can serve as a legitimate starting point for further analysis and knowledge generation

The MEB^3^ is an approach that proposes parallels where indigenous, local, and scientific knowledge systems are viewed to generate different manifestations of valid and useful knowledge. Through complementarities, different knowledge systems can contribute to an enriched picture as outlined in Fig. 1. The analysis of the enriched picture, including complementarities, synergies, and contradictions across diverse knowledge systems, can enhance the understanding of environmental conditions and change and the potential for sustainable management of ecosystems. The MEB highlights the importance of indigenous and local knowledge systems on their own terms, where evaluation of knowledge as useful and relevant for the issue of investigation occurs primarily within rather than across knowledge systems. It also recognizes differences within types of scientific knowledge and forms of evidence, such as between disciplines of natural and social sciences, or qualitative and quantitative approaches. Brought together through a collaborative process, multiple evidence on a common issue (e.g., Arctic sea ice dynamics, pollination services, or assessment programs such as in the IPBES) creates an enriched picture of understanding in an assessment process. The enriched picture has potential to widen the scope, depth and value of the assessment, and is also a starting point for further knowledge generation, within or across knowledge systems through cross-fertilization and co-production of knowledge. The process may also enhance the legitimacy and relevance of the assessment outcomes for a wide range of actors.

The MEB approach will be further elaborated below after a brief introduction to approaches for creating synergies across knowledge systems and key challenges involved. We continue with a discussion of scale and conclude by pointing out some key challenges and potentials for a MEB approach, in particular in local to global knowledge-policy processes such as the IPBES or the SDG.

## Approaches for Creating Synergies Between Knowledge Systems

As a starting point for any discussion of connecting knowledge systems, it is essential to keep in mind that different knowledge systems have always cross-fertilized and benefitted from each other and have rarely developed in isolation. However, in the context of knowledge-policy processes such as the IPCC or the IPBES, where power inequities and epistemological differences between diverse knowledge systems are brought to the fore, it is important to differentiate among (a) integration of knowledge, (b) parallel approaches to developing synergies across knowledge systems, and (c) co-production of knowledge.

Integration has been used differently by different scholars (see discussion in Stephenson and Moller 2009); here we emphasize processes that attempt to incorporate components of one knowledge system into another through a validation process based on the latter system (e.g., Gratani et al. (2011), where traditional fishing poisons for invasive fish management were evaluated using scientific laboratory trials). Scientific validation of traditional or local knowledge is often a more or less explicit requirement for inclusion of other knowledge systems (Agrawal 1995). This one-way process has been questioned for a number of reasons, such as whether the validation measures used are appropriate, exclusion of relevant and locally legitimate knowledge, and disempowerment of local communities (Nadasdy 1999; Nakashima and Roué 2002). In contrast, a parallel approach emphasizes complementarities while presupposing validation across knowledge systems (Agrawal 1995; Nadasdy 1999; Berkes 2008). Berkes (2008) writes on science and local knowledge that “each is legitimate in its own right, within its own context; each has its own strengths. The two kinds of knowledge may be pursued separately but in parallel, enriching one another as needed.” For example, Moller et al. (2004) compile evidence of population dynamics of hunted populations based on scientific publications and monitoring techniques, and local hunters’ accounts and practices respectively, see more details and further examples in Table 1.

Lastly, co-production of knowledge entails engaging in mutual processes of knowledge generation at all stages of knowledge generation, such as for example an assessment, including validation (Berkes 2008; Pohl et al. 2010; Rist et al. 2011; Shirk et al. 2012). Co-production of knowledge is part of many cases of co-management (e.g., Armitage et al. (2011)), community-based management (e.g., Ballard et al. (2008)), and participatory natural resource monitoring (Danielsen et al. 2009). Each of these three approaches may have their merit in a particular context, depending for example on the issue at hand, scale, and the past history of interactions between knowledge systems. For example, Gratani et al. (2011) show that integration of traditional knowledge through scientific validation can be respectful and empowering. It appears that the critical issue is the nature of the interactions among knowledge systems and that all involved are part of a collaborative process to determine which approach is the desirable. Box 1 elaborates on key attributes that frame mutually successful interactions between diverse knowledge systems, based on insights from a series of cross-cultural workshops on connecting knowledge systems. In this paper, we explore the MEB as a parallel approach, which we argue is a useful way to navigate the tensions involved in developing synergies between knowledge systems.

**Box 1:** Dialogue workshop on knowledge for the twenty-first century in Guna Yala, Panama

The workshop brought together respresentatives from a diversity of knowledge systems including local and indigenous knowledge, social and natural science, as well as NGOs and decision makers. While there are many different approaches for exchange, it was found that the attitudes framing the interactions are essential, such as *respect*, *trust*, *reciprocity* and *equal sharing.* Among key factors for success was the recognition that learning and knowledge may relate to spiritual belief systems^a^	
On validation, the workshop recognized that indigenous and local knowledge systems have their own internal systems achieving empirical and social legitimacy of knowledge and hence its validation. These may include experimental and empirical as well as experiential validation based on cultural norms and historical experiences through experiments, expert peer-review, and collective procedures for evaluating and cross-examining knowledge including mechanisms for intergenerational transmission of knowledge	
It was argued that validation mechanisms need to be aligned with the knowledge system it aims at representing. For example, reductionist requirements of hypothesis testing cannot be used to validate knowledge generated within a systems or relational based knowledge systems, as this would fail to recognize emergence or holistic aspects. The workshop concluded that for the purpose of IPBES we need to look further into validation mechanisms that recognize diverse knowledge systems using separate protocols, as developed through respectful intercultural dialogue. Based on report edited by Tengö and Malmer (2012), see also www.dialogueseminars.net/panama	

^a^See Rist et al. (2011) for an elaboration

### Validation and Evaluation of Knowledge

Validity is a term with multiple interpretations that applies to instruments of data collection (i.e., whether purely observational or written or mechanical/electronic), to the type of data collected by such instruments (i.e., whether qualitative or qualitative, measured or interpreted), to the type of finding derived from the data (i.e., the basis of judgment used for data assessment), and to the kind of explanation derived from the interpretation of findings (i.e., the different viewpoints and contexts used in interpretation). Furthermore, reliability and level of precision—two criteria from which to judge instrument, data, and explanation—may vary within each of these domains and ultimately depend on determining what is considered and who should consider the best judgment of a given phenomenon.

In different knowledge systems, or branches within a system, the criteria and methods to validate knowledge can differ significantly (Schweizer 2006). For example, quantitative research within the natural and social sciences relies on specific sampling designs and/or repeatable experiments and results, whereas qualitative research in the social sciences and humanities may use different approaches to generate and to validate data, stressing in particular attention to the social context associated with a given analysis. In addition, validation problems commonly emerge in the process of generalizing information across scales, whereas context-specific knowledge loses meaning when applied to other situations.

The challenge of validation has been a historical problem for the social sciences. Positivistic, humanistic, phenomenology, and hermeneutic approaches to the understanding of human behavior and thoughts have disputed not only whether the objectivity of the physical sciences applies to human societies, but what conceptual constructs and instruments are relevant (Bernard 2011). The notion of validity is at the very intersection of these tensions; as mentioned above, it depends on the kinds of observational tool one uses to collect “data” and on the collective judgment that makes sense of a phenomenon through such observations. While fundamental philosophical differences exist regarding the nature of human behavior, thought, and affairs, often disagreement emerges out of different types of conceptual [mis]understandings, i.e., for the purpose of this article, “validity mismatches.”

In the realm of human–environment interactions, the issue of interest may vary in nature and complexity, whereas the level of match and mismatches between approaches (within the sciences and between science and other knowledge systems) can range from close agreement (such as on estimating the agro-ecological diversity of a garden) to complete dissonance and conflict (such as on interpreting the cultural meaning and significance of such agro-ecological diversity and why it is esthetically arranged in a particular way within a garden). Thus, the nature of validation and potential for collaboration across and within knowledge systems vary according to the nature of the issue or problem at hand. As commonly experienced in research that crosses disciplinary divides, using the validation methods of one certain system (e.g., quantitative natural science) to validate knowledge from other systems (e.g., quantitative and/or qualitative social science or indigenous knowledge systems), may lead to compromising the quality or integrity of the latter knowledge, and the potential rejection of valid knowledge. For example, Fortmann and Ballard (2011) argue that that overly narrow understanding of what constitute valid scientific practice have led to the detrimental exclusion of knowledge produced by local scientific practices from official forest management and forest policy in the US. In the context of interaction between science and other knowledge systems, the problem extends from validation of knowledge to mistrust about the legitimacy of knowledge, particularly in the context of policy-related processes. In other words, a failure to capture claims and perspectives of different knowledge holders in policy-related processes may undermine the participation of different groups in decision making as well as the perceived legitimacy of the outcomes of such processes (Agrawal 1995).

Conceptual frameworks have increasingly been used as tools for inter- and trans-disciplinary collaboration and as a way to overcome some of the limitations above. Ostrom (2011) for instance suggests that conceptual frameworks facilitate the integration of different types of theories and models as they apply to different questions and parts of a research problem, but together contribute to the understanding of a whole. A MEB approach builds upon these efforts by calling attention to the importance of bringing together multiple knowledge systems in an equal and transparent way.

MEB stresses the importance of grounding collaborations on an equal starting point whereas contributors define the goals of the collaboration and mutually agreed ways to proceed. In other words, for potential synergies across knowledge systems, processes for validating knowledge need to recognize and respect differences in theoretical and methodological approaches to understanding the biophysical world as well as the underlying worldviews (Lyver et al. 2009; Brondizio et al. 2010; Bohensky and Maru 2011). This implies a process of “intersubjectivity” where there is common understanding about not only the scope of the collaboration, but also of the meanings and complementarities of types of observations and judgments. In this sense, validity is interpreted here not only as the extent to which our observations reflect the phenomena we are interested in (which implies continually checking, questioning and theoretically interpreting findings), but the collective judgment we can derive from such interpretations. For example, one can use different data sources to triangulate, checking the meaning of extreme cases, looking for contrary examples, checking for rival explanations, and obtaining feedback from collaborators. This “intersubjective” approach to collaboration across knowledge systems should be complemented by “communicative validity,” in which the validity of knowledge claims is tested in a dialogue with informants and peers (Kvale 1995). Box 1 provides insights on validation as generated in a cross-cultural workshop.

## The Multiple Evidence Base Approach

A MEB approach emphasizes the complementarity of knowledge systems and the values of letting each knowledge systems speak for itself, within its own context, without assigning one dominant knowledge system with the role of external validator. Complementary insights from different knowledge system create an enriched picture of a case study or the broader issue of investigation. A first proposal for a MEB approach was presented in a report from the International Science Workshop on Assessments for IPBES.^4^ “The [multiple] evidence-based peer-review process takes into account that different criteria of validation should be applied to data and information originating from different knowledge systems. ‘[Multiple] evidence-base’ means that in the assessments, the different knowledge systems are viewed as generating equally valid evidence for interpreting change, trajectories, and causal relationships.” Here, we develop the approach of bringing together multiple evidence, drawing on numerous discussions with representatives from diverse knowledge systems, including indigenous and local knowledge systems as well as natural and social sciences, following presentations, sharing and reflections in meetings and workshops in the context of the CBD and the IPBES.^5^

The approach acknowledges that there are power issues involved when connecting different branches of science with locally based knowledge systems (Agrawal 1995; Nadasdy 1999; Derkzen and Bock 2007), and that there are—despite similarities and overlaps—aspects of each knowledge system that cannot be fully translated into another (Tengö and Malmer 2012). Parallel approaches emphasizing complementarity are proposed by many scholars on indigenous and local knowledge, as well as are embedded in on-going cross-cultural practice across the world (Turnbull 1997; Berkes 2008; Moller et al. 2009; Rist et al. 2011; Haverkort et al. 2012). The MEB approach aims to promote and enable connections across knowledge systems in a respectful and equal manner. The approach stresses that the type of complementarity and co-production envisioned should be part of a collaborative process between those involved from the onset. The focus on the process may help to leverage the power dynamics, maintain integrity of knowledge systems, generate new questions, and thus enable ecosystem assessments and knowledge generation that are salient, credible, and legitimate for knowledge holders at different scales (Cash et al. 2003; Reid et al. 2006).

Table 1 presents examples of case studies where collaborative approaches to connecting knowledge systems have been applied, coherent with a MEB approach. The cases were selected to represent clear examples based in a parallel approach to connecting knowledge systems, from a range of different resources or ecosystems in different parts of the world. As we were looking for examples published in scientific literature, most of them are science driven. However, in the science-practice realm there are many emerging initiatives that use approaches similar to a MEB, see for example Danielsen et al. (2009, 2014), Rist et al. (2011), and Shirk et al. (2012), see also Box 2. The cases in Table 1 illustrates the potential complementarity of knowledge systems in terms of spatial and temporal scales, understanding species and ecosystem diversity, as well as understanding the drivers and processes of social and environmental change. In some cases, local knowledge offers fine grain information about particular phenomena (e.g., sea ice change, fish school movements, and forest management techniques) while in other cases, it helps to extend the spatial (e.g., reindeer herding using a landscape scale for understanding biodiversity changes) and temporal (e.g., key historical events or oral history expanding the time depth) scales of observation. In other cases, local knowledge contributes to understanding how macro drivers of change interact with local drivers, thus complementing science in terms of scaling up the outcomes of such interactions. Moller et al. (2004) point out that local knowledge has a strength in identifying relevant hypotheses for problem solving, which is complemented by powerful tools of science to address and evaluate the underlying mechanisms involved.

**Box 2:** Community-based monitoring and information systems (CBMIS)

CBMIS is a joint initiative among a global network of indigenous peoples and local communities, which seeks to combine the monitoring needs of communities with needs for detailed data as a base for joint action related to territories and resources (CBD 2013b; Stankovich et al. 2013). CBMIS aims at assessing the state of indigenous and local knowledge, biodiversity, climate change impacts, and community well-being, and is now being piloted and developed by a network under the International Indigenous Forum on Biodiversity Working Group on Indicators, together with many collaborators. The monitoring contributes to strengthen the local knowledge base for territorial resource management and community development, as well as contributing case studies and complementary data for monitoring the Convention on Biological Diversity Strategic Plan for Biodiversity and Aichi Targets and other international commitments under climate change and sustainable development. This same approach could also be important in contributing to the IPBES functions of assessments, knowledge generation, policy relevant tools and capacity building (CBD 2013a)	

Examining the enriched picture using a MEB approach can enable triangulation of information across knowledge systems and evaluation of the relevance of knowledge and information at different scales and in different contexts. As pointed out by Chalmers and Fabricius (2007), it is important to acknowledge and recognize the power issues and inequalities involved in relating for example local knowledge and scientific knowledge to each other, ensuring that triangulation is multidirectional. This further emphasizes the importance of a process that promotes participation through all stages of collaboration (see also Rist et al. (2011)). An understanding based on multiple evidences can enable stronger confidence in conclusions where knowledge and understanding converge across knowledge systems. It may also highlight complementarities or disagreements that in turn may generate new insights or hypotheses and ideas to study further (Moller et al. 2004; Gagnon and Berteaux 2009).

In processing the enriched picture, conflicting or contradictory evidence should not be neglected or concealed but accepted as such since there is some knowledge and information that will remain incompatible. The diversity of perspectives can benefit further knowledge generation as well as decision making. The enriched picture creates an opportunity for “a culturally informed appraisal of scientific knowledge and practice so as to differentiate between elements that could be recognized as ‘universal’ or shared among knowledge systems as opposed to ‘relative’ or unique to a specific knowledge system” (IPBES 2013b). An outsider’s perspective through cross-cultural peer-review can be mutually beneficial (Stephenson and Moller 2009). For example, it has been shown that combining scientific and local methods for monitoring wildlife provides, on the one hand, an opportunity for customary users to scrutinize science and, on the other hand, for science to learn about relationships and processes previously unknown (Moller et al. 2004; see also Prado et al. 2013 for complementarity between local and scientific knowledge of wildlife). In addition to enhancing the relevance of knowledge used for decision making, it increases trust and avoids the arrogance of a single “right approach” commonly represented by science (Mackinson 2001; Moller et al. 2004).

If representatives from diverse knowledge systems, including scientists and decision makers, accept each other’s legitimacy and power, space is created for developing collaboration from the onset of a project, grounded on the appreciation of different ways of understanding the world. Empowered and respectful partnerships are a constructive starting point to investigate and identify solutions for environmental change and sustainable development (Lyver et al. 2009; Rist et al. 2011).

### Multiple Evidence Base in the IPBES

**Fig. 2:**
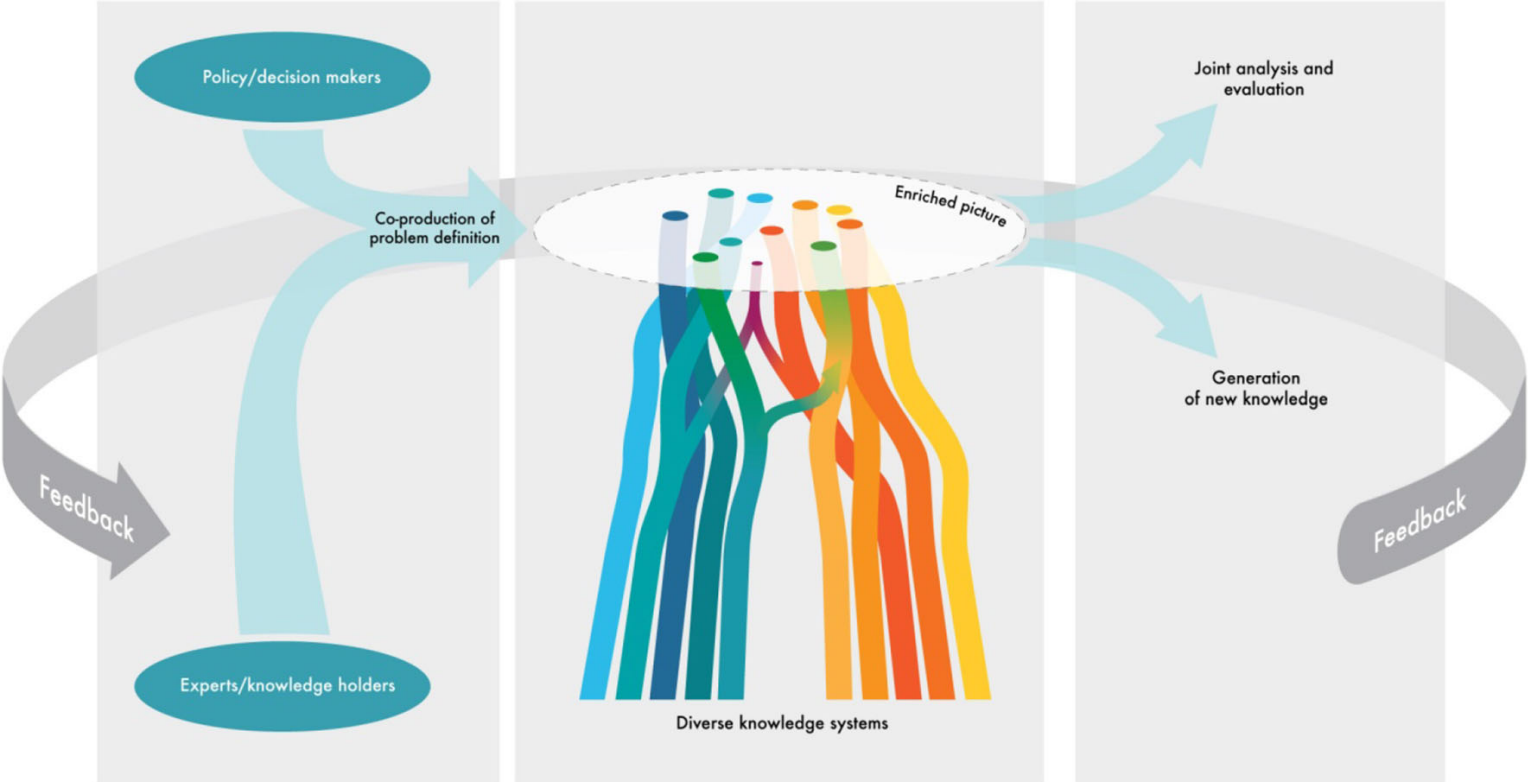
Outlining three phases of a multiple evidence base approach that emphasizes the need for co-production of problem definitions as well as joint analysis and evaluation of the enriched picture created in the assessment process. *Phase 1* involves defining problems and goals in a collaborative manner that recognizes cross-scale interactions of drivers and local responses and sets the stage for maintaining ongoing dialogue. This includes establishing partnerships between relevant communities, organizations and networks as appropriate and needed at different levels; investigating common interests and concerns, including power relations among actors; recognizing differences in experiences, methods, and goals across actors (Laidler 2006). *Phase 2* involves bringing together knowledge on an equal platform, using parallel systems of valuing and questions and domains. This includes acknowledging and recognizing the spatial and temporal context of knowledge and implications for scalability; acknowledging and addressing power issues among knowledge systems and holders; consideration of different areas of strength and contribution of different knowledge systems and their overlaps; and acknowledging converging and diverging evidence and perspectives across knowledge systems. *Phase 3* involves joint analysis and evaluation of knowledge and insights to generate multi-level synthesis and identify and catalyze processes for generating new knowledge. This includes identifying continuing knowledge gaps, new hypothesis, and potential areas for new collaborations across knowledge systems. To enable these processes, there is a need to develop new tools and approaches for combining and relating multiple data, including qualitative as well as quantitative

Figure 2 outlines a MEB as three basic stages for consideration by assessment programs such as the IPBES. First, it emphasizes the importance of defining problems and goals in a collaborative manner. While the plenary and the Multidisciplinary Expert Panel (MEP) set priorities for the IPBES, the onset of a particular activity, such as assessments, must involve those who live from and directly depend upon the resources or ecosystems for their livelihoods. Since the IPBES activities may or may not include clearly defined geographical areas, it may require the involvement of different kinds of knowledge holders and experts at different levels of analysis. For example, a thematic assessment of pollination and food production as set out in IPBES Work Programme 2014–2018 (IPBES 2013b) will substantially gain from engagement with knowledge holders among bee keepers and honey gatherers and different groups of farmers irrespective of the continent, but in particular from Africa, Asia and Latin America, because of the limited amount of studies available from these areas. At this stage and given the predominant regional scale of IPBES assessments, the elements of a “nested approach” should be considered, as discussed in “Multiple Evidence Base and Scale” section below.

While challenging, the co-production of problem or goal definition is particularly important to create a collaborative platform for synergies across knowledge system. It is also important for the development of an institutional culture that accepts the inherent complexity of issues related to assessment programs such as the IPBES. The second stage of an MEB oriented assessment process sets forward the generation of an enriched picture of the problems and goals defined in the first stage, drawing on an agreed upon diversity of knowledge. Similarities, complementarities, as well as contradictions across knowledge systems can be evaluated and discussed, and form the basis for a final assessment as well as further knowledge generation. Finally, in the third stage, the parties involved should consider and reflect on the social and environmental implications of results, including a re-assessment of knowledge gaps and new opportunities for collaborative activities. The assessment process should in itself be evaluated as part of a constructive and cumulative learning process.

To develop synergies across knowledge systems, as a cross-cutting issue within the structure of the IPBES or similar bodies or in specific assessments or projects, requires continuous dialogue during all stages of the process (Rist et al. 2011). According to Yankelovich (1999), there are three distinctive features that differentiate a dialogue from a discussion. When all three are present, a conversation is transformed into a dialogue: equality and the absence of coercive influences, listening with empathy, and bringing assumptions into the open. It is important to create forums representative of different sub-areas within a region in which multiple knowledge holders and/or practitioners can exchange knowledge and experiences. However, power relations between the various participants need to be taken into account. It is all too often assumed that such forums are neutral spaces in which all participants can express themselves and be heard (Edmunds and Wollenberg 2001). Yet, without specific attention to disadvantaged groups, there is a risk that those considered “experts” will dominate the debate (Derkzen and Bock 2007). The focus on obtaining consensus, which is often assumed to be a prerequisite for these kinds of forums, may lead to the false impression that all participants share the “experts” views (Edmunds and Wollenberg 2001; Voß and Bornemann 2011).

Furthermore, conflicts between different knowledge holders and/or practitioners may not actually be about conflicting evidence, but about controversial policy decisions that may be masked as evidence issues (Voß and Bornemann 2011). It is therefore important to acknowledge that evaluating biodiversity, ecosystem services, and more broadly environmental change, is inherently political and concerns trade-offs and different interests. This includes the local level where the often more vocal and politically articulated leaders may obscure the voices of others. This needs to be addressed explicitly, taking into account the distribution of costs, risks, and benefits of specific policy options across and between different stakeholder groups (Voß and Bornemann 2011; Spierenburg 2012).

### Multiple Evidence Base and Scale

In assessment processes, addressing multiple scales and paying explicit attention to social and environmental heterogeneity within each scale are essential. A multi-scalar approach contributes to understanding the complex and diverse local and sub-regional effects of national and global drivers of change, from climate change to market fluctuations, as well as local responses to change. Furthermore, it is also required to identify areas where local actors can improve their situation by taking advantage of emerging opportunities or buffer potentially negative impacts of macro-level drivers.

The type and level of complementarity across knowledge system will vary according to context, the issue addressed, and the desired outcomes. The scale of observation that forms the basis for different knowledge systems is critical when evaluating the congruence between them for a given subject (Gagnon and Berteaux 2009). Studies, such as those represented in Table 1, show that different knowledge systems often are complementary in terms of which scale they focus on, and that the combination of approaches leads to better understanding of cross-scale interactions (Laidler 2006; Gagnon and Berteaux 2009). As illustrated in Fig. 2, the process of defining the goals, however, is fundamental to avoid a mismatch of expectations.

Furthermore, scale also matters in the definition, collation, compilation and aggregation of knowledge both horizontally, e.g., across local communities, and vertically; in other words the implications for scaling knowledge up and down for decision making (Reid et al. 2006). New methods are needed to find innovative ways for legitimate and constructive ways of aggregating, evaluating, and synthesizing knowledge to inform scales beyond the local (Berkes et al. 2006). Brondizio (2008) provide an example of an approach for linking site specific knowledge and landscape-level analysis. Geographical centers for compiling knowledge and insights, similar to the Satoyama/Satoumi process of articulating knowledge within and across different regional “clusters” within Japan (Japan Satoyama Satoumi Assessment 2010) using a MEB approach may be part of a solution. The Millennium Ecosystem Assessment developed a framework that allowed for the use of a wide range of indicators (Pereira et al. 2005) although these still lacked integration and were not scalable. Indicators were originally designed to span national to global scales but it has been repeatedly emphasized that a set of scalable indicators is needed, which could be used for the upscaling of observations from local to global scales as well as downscaling (Scholes et al. 2013). However, there are tensions between finding indicators that are comparable across regions and indicators that are sensitive to local priorities and hence relevant to local stakeholders (Mitchell and Parkins 2011). The community-based monitoring and information systems (CBMIS) is a bottom-up approach to develop indicators that make sense on a local scale and jointly explore the potentials to scale up on a regional and even global scale, see Box 2. This opens up the possibility to engage diverse knowledge holders in monitoring, analyzing, and reporting, and the approach has received attention within the CBD as well as IPBES (CBD 2013b).

Separating knowledge from its local, cultural, and epistemological context can involve significant risks for indigenous peoples and local communities (Agrawal 2002). Williams and Hardison (2013) call for safeguards related to communities’ rights to their knowledge, including proper implementation of Free Prior Informed Consent (FPIC) related to sharing of their knowledge, and capacity building on the potential risks. This is a critical issue to be addressed in for example the IPBES.

## Conclusions

Transforming governance of biodiversity and ecosystems toward sustainability will require a rich understanding of the complex interactions of people and nature at different scales, and of the drivers and feedbacks that affects these interactions. With new challenges in a rapidly evolving human-dominated world, we need to nurture the broad range of sources of knowledge and learning to be able to deal with global environmental change and new social–ecological conditions. We argue that to achieve this, the science-policy community needs to embrace a diversity of knowledge systems, and when connecting to knowledge from local or indigenous communities, it must think beyond aspects that can easily be fitted into conventional models and frameworks. Recognition of these knowledge systems’ capacity to underpin, maintain, and generate new understandings of dynamic ecosystems and changing social–ecological conditions is essential (Berkes 2008). Furthermore, there is a need to acknowledge, respect, and involve experts from diverse knowledge systems into assessments and other knowledge related processes, as well as in developing the procedures for *how* to design such processes. We also need to develop methodologies and approaches to link and build complementarities of knowledge systems across scales. Our vision for a MEB approach is to contribute to a mind shift toward such recognitions across a diversity of scales and processes and encourage new collaborative efforts.

International programs and bodies, such as the CBD, the IPBES, and the development of the new Sustainable Development Goals have a clear ambition to build on insights from diverse knowledge systems. We see the development of a MEB approach within such efforts, such as in the recommendations from the CBD Eight Working Group Meeting on Article 8(j) and Related Provisions (CBD 2013a) and, e.g., the IPBES conceptual framework (IPBES 2013a), as a promising step in the right direction.

The following are key challenges to be addressed in developing a MEB in the context of assessment programs and monitoring of the SDGs:

- *Fundamental values*. It is important to establish frameworks to promote and enable equal and transparent connections between knowledge systems, to level the power dynamics involved, to empower communities, and also fulfill the potential of knowledge synergies for ecosystem governance. Fundamental values such as respect, trust, reciprocity, and equal sharing need to characterize all interactions at all scales (Tengö and Malmer 2012). To enable successful synergies across knowledge systems, there is a *need for true intercultural dialogues*, which gives and promotes credibility and legitimacy for those involved (Yankelovich 1999; Cash et al. 2006; Rist et al. 2011). The MEB is an approach for generating the levels of trust and respect required for dialogues leading to changing mental models and widened perceptions of how knowledge systems can cross-fertilize among all knowledge holders. For example, a development of parallel sets of validation criteria for diverse knowledge in for example the IPBES needs to be based on an inclusive and transparent dialogues.
- The *development of procedures* concerning the problem definitions, the assessment process, and the evaluation of findings needs to involve co-production and collaboration with relevant stakeholders from the onset (Zingerli 2011). A particular challenge for programs such as IPBES is to engage and promote representation of different stakeholder groups within large geographical regions. While challenging, the co-production of problem or goal definition is particularly important to create a lasting collaborative platform for synergies across knowledge systems. It is also important for the development of an institutional culture which accepts the inherent complexity of human–environment related issues.
- A key challenge is making indigenous and local knowledge *matter at scales beyond the local* (Reid et al. 2006) while avoiding loss of legitimacy among knowledge holders as well as decision makers at different levels. Local responses to environmental changes can mediate or reinforce global dynamics, and cross-scale interactions need to be better understood to support and encourage stewardship of the biosphere (Folke et al. 2011). For example, the task of developing more integrated and scalable indicators will be crucial for the Aichi targets of the CBD and Sustainable Development Goals, since it is important to base information on the results of localized interactions (see, e.g., CBD 2013b). Using indicators that make sense on a local scale opens up the possibility to engage local stakeholders, citizen groups, indigenous groups and many other knowledge holders in the monitoring, reporting and development of the goals as well as in the process of potentially scaling up the indicators (Danielsen et al. 2014). The MEB approach could facilitate this development into a set of robust and agreed upon scalable indicators.
- A MEB approach emphasizes *the value of diversity* and recognizes a multitude of ways to address the challenges of cross-fertilization that are firmly rooted in some key principles that are agreed upon by all parties. It should be viewed and recognized as a process which is considered in relation to different goals, geographical regions, and needs as expressed by the actors involved. There is great potential to find mechanisms for learning across “success stories” of synergies between knowledge systems, while still adjusting for contextual factors (Stephenson and Moller 2009; Rist et al. 2011; Danielsen et al. 2014, see also Table 1).
- *Need for new methods* there is a need for new tools and approaches for co-production of questions and issues, methods for mobilizing, documenting and sharing knowledge for the enriched picture, as well as methods for the co-production of analyses and insights based on the enriched picture (see Fig. 2). This is not only needed to facilitate collaboration between local and scientific knowledge, but also between types of scientific knowledge, such as between the natural and social sciences and the humanities, as well as between quantitative and qualitative approaches within or across disciplines. Some examples are the use of modeling tools such as fuzzy logics or approaches such as Bayesian statistics (Mackinson 2001; Berkes and Berkes 2009). Sutherland et al. (2013) suggests consensus methods such as the Delphi technique to combine knowledge from multiple sources of evidence. Programs and bodies such as the IPBES offer excellent opportunities to develop such new methods. In its adopted Work Programme 2014–2018, the IPBES has established a Task Force to advancing procedures and approaches for indigenous and local knowledge (IPBES 2013b).

To conclude, whether as part of assessments, e.g., in the IPBES, monitoring of the SDGs, or local ecosystem management projects, we view connecting knowledge systems through a MEB approach not only as a way to mobilize existing knowledge for assessments and improved policy, but also as a way to support and enhance mechanisms for learning and decision making in the context of dynamic social–ecological systems. We see it as a way to nurture a diversity of sources for experience, insights, and innovations for sustainable governance of ecosystems and biodiversity in the Anthropocene, which embraces respect, reciprocity and equity in the social learning processes across diverse knowledge systems.

## Footnotes

1 We refer to knowledge systems as made up of agents, practices, and institutions that organize the production, transfer and use of knowledge (Cornell et al. 2013).

2 The same applies to knowledge from practitioners and ecosystem managers, although not covered in this paper.

3 The development of thinking around "evidence-based” and “evidence-based practice” has a long history, particularly in medicine and was formally defined in medicine by Sackett et al. (1996) as, “the conscientious and judicious use of current best evidence from clinical care research in the management of individual patients.” The Cochrane Collaboration (www.cochrane-net.org) have resulted in that evidence-based medicine has now become a global concept, including systematic reviews sometimes based on different epistemological frameworks and subject to, e.g., a Bayesian synthesis of disparate data types. Evidence-based natural resource or conservation management is given a lot of attention in recent years, but sofar it is only building on western scientific knowledge systems, see for example www.conservationevidence.com and www.environmentalevidence.org or Mascia et al. (2014).

4 Workshop report, International Science Workshop on Assessments for IPBES, United Nations University, Tokyo, Japan. 25–29 July 2011.

5

- “*Dialogue workshop on Knowledge for the twenty-first century: Indigenous knowledge, traditional knowledge, science and connecting diverse knowledge systems.*” Usdub, Guna Yala, Panama, organized by SwedBio at Stockholm Resilience Centre, International Indigenous Forum on Biodiversity and Naptek at the Swedish Biodiversity Centre from 10 to 13 April 2012.
- “4th Sub-Global Assessment Network Annual Meeting,” including the session on “*Indigenous, Traditional and Local Knowledge in Assessments*,” organized by UNEP-WCMC and the Cropper Foundation in Stellenbosch, South Africa from 26th to 29th November 2012
- *“Global Planning Workshop on Community-Based Monitoring and Information Systems”,* organized by Tebtebba Foundation, in Quezon City, Philippines from 28 February to 1 March 2013;
- “*International Expert Workshop connecting diverse Knowledge Systems in the context of IPBES”,* organized by the German Federal Agency for Nature Conservation (BfN) in cooperation with the Institute for Biodiversity Network e.V. (ibn), at the International Academy for Nature Conservation, Isle of Vilm, Germany from 22 to 25 April 2013;
- “*Global Expert Workshop on Community-based Monitoring and Information Systems*”, organized by Tebtebba Foundation, the Forest Peoples Programme, the Indigenous Peoples’ Partnership on Climate Change and Forests, the International Indigenous Forum on Biodiversity’s Working Group on Indicators, the Article 10(c) customary sustainable use network, SwedBio at Stockholm Resilience Centre and the Secretariat of the Convention on Biological Diversity, held in Bonn, Germany, from 26 to 28 April 2013.
- “*World Indigenous Network (WIN) Conference*”, organized by the Government of Australia, with other partners including Brazil, Canada, Norway, and New Zealand, including the workshop session “*Connecting indigenous, traditional and local knowledge and science—such as in IPBES—what´s in it for knowledge holders?*” held in Darwin, Australia from 26 to 31 May, 2013.
- “*The Eight Meeting of the Ad Hoc Open Ended Working Group on Article 8(j) and Related Provisions of the Convention on Biological Diversity*,” including its In Depth Dialogue on “*Connecting Traditional Knowledge Systems and Science, such as under the IPBES, Including Gender Dimensions*", held in Montreal, Canada from 7 to 11 October, 2013.
